# E2F1 Regulates Adipocyte Differentiation and Adipogenesis by Activating ICAT

**DOI:** 10.3390/cells9041024

**Published:** 2020-04-21

**Authors:** Jingqing Chen, Yuchen Yang, Shuai Li, Ying Yang, Zhaolai Dai, Fengchao Wang, Zhenlong Wu, Patrick Tso, Guoyao Wu

**Affiliations:** 1 State key Laboratory of Animal Nutrition, China Agricultural University, Beijing 100193, China; CJQ9512@163.com (J.C.); yuchen_yang992@163.com (Y.Y.); l-shuai@outlook.com (S.L.); cauvet@163.com (Y.Y.); daizhaolai@cau.edu.cn (Z.D.); 2 National Institute of Biological Sciences (NIBS), Beijing 102206, China; wangfengchao@nibs.ac.cn; 3 Beijing Advanced Innovation Center for Food Nutrition and Human Health, China Agricultural University, Beijing 100193, China; 4 Department of Pathology and Laboratory Medicine, Metabolic Diseases Institute, University of Cincinnati, Cincinnati, OH 45215, USA; tsopp@ucmail.uc.edu; 5 Department of Animal Science, Texas A&M University, College Station, TX 77843, USA; g-wu@tamu.edu

**Keywords:** adipogenesis, differentiation, E2F1, ICAT, Wnt/β-catenin, 3T3-L1

## Abstract

Wnt/β-catenin is a crucial repressor of adipogenesis. We have shown that E2 promoter binding factor 1 (E2F1) suppresses Wnt/β-catenin activity through transactivation of β-catenin interacting protein 1 (CTNNBIP1), also known as inhibitor of β-catenin and TCF4 (ICAT) in human colorectal cancers. However, it remains unknown whether ICAT is required for E2F1 to promote differentiation by inhibiting β-catenin activity in pre-adipocytes. In the present study, we found that 1-methyl-3-isobutylxanthine, dexamethasone, and insulin (MDI)-induced differentiation and lipid accumulation in 3T3-L1 pre-adipocytes was reversed by activation of β-catenin triggered by CHIR99021, a GSK3β inhibitor. Intriguingly, we observed a reduced protein level of E2F1 and ICAT at a later stage of pre-adipocytes differentiation. Importantly, overexpression of ICAT in 3T3-L1 pre-adipocytes markedly promote the adipogenesis and partially reversed the inhibitory effect of CHIR99021 on MDI-induced adipogenesis and lipid accumulation by regulating adipogenic regulators and Wnt/β-catenin targets. Moreover, pre-adipocytes differentiation induced by MDI were markedly inhibited in siE2F1 or siICAT transfected 3T3-L1 cells. Gene silencing of ICAT in the E2F1 overexpressed adipocytes also inhibited the adipogenesis. These data indicated that E2F1 is a metabolic regulator with an ability to promote pre-adipocyte differentiation by activating ICAT, therefore represses Wnt/β-catenin activity in 3T3-L1 cells. We also demonstrated that ICAT overexpression did not affect oleic acid-induced lipid accumulation at the surface of Hela and HepG2 cells. In conclusion, we show that E2F1 is a critical regulator with an ability to promote differentiation and adipogenesis by activating ICAT in pre-adipocytes.

## 1. Introduction

The alarming increase in the incidence of obesity among adults and children worldwide has prompted extensive researches on molecular mechanisms responsible for the synthesis and catabolism of triglycerides (TG) in white adipose tissues [1]. A compelling evidence shows that an excessive accumulation of fat mass in obese subjects is associated with an increase in adipocyte volume (hypertrophy), number (hyperplasia), or a combination of both (hypertrophy–hyperplasia) [2]. It is generally believed that the increased number of adipocytes is mainly determined by the adipocyte differentiation process, termed adipogenesis [3]. Adipogenesis consists of a complex series of events in which both cellular and extracellular factors act together and lead to transformation to a mature, lipid-filled adipocyte from a fibroblast-like pre-adipocyte. Therefore, pre-adipocyte differentiation has been one of the well-known models used to study adipogenesis [4], and underlying mechanisms, due to its implication in metabolic syndrome, such as insulin resistance, type 2 diabetes, hypertension, and atherosclerosis [5].

E2 promoter binding factor 1 (E2F1) is a transcriptional factor involved in cell cycle progression, cell differentiation, and apoptosis [6]. Several lines of studies show that E2F1 is a novel regulator of metabolic homeostasis [7,8,9]. Protein level of E2F1 is increased in the visceral white adipose tissue of obese human subjects and is positively correlated with development of insulin resistance, circulating free fatty acids level, and incidence of non-alcoholic fatty liver disease [7,10,11,12,13]. In contrast, E2F1^−/−^ mice have a reduced fat accretion, increased insulin sensitivity, and a decreased circulating level of cholesterol [14,15,16]. Additionally, knockout of E2F1 in pre-adipocytes impairs its capacity to differentiate into adipocytes [14], indicating a critical role of E2F1 in differentiation. These studies highlight an unexpected functional role of E2F1 on cellular metabolism in both humans and animals. However, it remains unknown how the transcriptional factor E2F1 interacts with regulators of pre-adipocyte differentiation and contributes to development of metabolic diseases.

In our previous study on E2F1-driven transactivation of downstream targets and its function in cancer cells, we have shown that inhibitor of β-catenin and TCF4 (ICAT), also known as the β-catenin interacting protein 1 (CTNNBIP1), is a direct transcriptional target of E2F1 [17]. Importantly, we found that activation of ICAT by E2F1 is required to inhibit β-catenin activity in colorectal cancers. An inhibitory effect of ICAT is mainly mediated by blocking the binding of β-catenin with T-cell factor 4 (TCF4), therefore leading to the repression of β-catenin-TCF4-mediated transactivation [17,18]. This finding has uncovered a link between Rb/E2F1 signaling with Wnt/β-catenin signaling pathway. In addition to functioning as an oncogene in various human malignancy, Wnt is an indispensable regulator for cell proliferation, survival, cell fate decision, and pre-adipocyte differentiation [19,20,21]. It has been reported that Wnt/β-catenin represses the expression of CCAAT/enhancer binding protein α (C/EBPα) and peroxisome proliferator-activated receptor γ (PPARγ), two critical adipogenic transcription factors involved in adipogenesis [19,22,23], thus implicated in adipocyte differentiation and metabolic diseases [22]. However, it remains unknown whether ICAT is also required for E2F1 to promote differentiation by inhibiting β-catenin activity in pre-adipocytes. In this study, we seek a better mechanistic insight into E2F1 regulation of adipogenesis by transactivating ICAT in pre-adipocytes.

## 2. Materials and Methods

### 2.1. Reagents

3T3-L1 pre-adipocytes, 293T cells, Hela, and HepG2 cells were obtained from the American-Type Culture Collection (ATCC, Manassas, VA, USA). Dulbecco’s Modified Eagle Medium (DMEM), Fetal Bovine Serum (FBS), and penicillin/streptomycin were obtained from Gibco BRL (Gibco BRL, Gaithersburg, MD, USA). Primary antibodies against β-catenin, PPARγ, C/EBPα, CCND1 (Cyclin D1), and c-MYC were purchased from Cell Signaling Technology (Beverly, MA, USA). E2F1 antibody was purchased from Santa Cruz Biotechnology (Santa Cruz, CA, USA). CTNNBIP1 (ICAT) antibody was purchased from Abcam (Cambridge, UK). Peroxidase-conjugated goat anti-rabbit and goat anti-mouse secondary antibodies were purchased from Huaxingbio Biotechnology Co (Beijing, China). The pIRES2-EGFP and pLent-EF1a-FH-CMV-GFP expression vector, packaging plasmid psPAX2 and envelope plasmid pMD2.G were all purchased from miaolingbio (Wuhan, China). BODIPY493/503, Lipofectamine RNAiMAX Reagent and Lipofectamine 3000 were purchased from Invitrogen (Carlsbad, CA, USA). CHIR99021 (GSK3β inhibitor), serum TG determination kit, insulin, dexamethasone (Dex), 1-methyl-3-isobutylxanthine (IBMX), oil red O, and all other reagents not indicated were purchased from Sigma Chemical Co. (St. Louis, MO, USA).

### 2.2. Cell Culture

Cells were maintained in DMEM supplemented with 10% FBS, 100 units/mL penicillin G sodium and 100 mg/mL streptomycin sulfate. Monolayer of 3T3-L1 pre-adipocytes were induced to differentiate into mature adipocytes as previously described [24]. Briefly, 2 days after post-confluence (designated as day 0), cells in 6-well plates were induced to differentiate by the addition of a standard cock-tail composed of 0.5 mmol/L IBMX, 1 μmol/L Dex, and 10 μg/mL insulin in the medium (designated as MDI cocktail). The differentiation medium was withdrawn 2 days later and replaced with medium supplemented with 10% FBS and 10 μg/mL insulin. After 2-day incubation, the cells were then cultured in a medium containing 10% FBS for another 3 days. During the differentiation process, CHIR99021 (0, 0.5, 1.0, 2.0, 3.0, or 4.0 μmol/L) was added in the differentiation medium for 7 days. All cell cultures were conducted at 37 °C in a 5% CO_2_ incubator. When 60–70% confluence was reached, cells were passaged using trypsin, and passages 5–15 cells were used in the present study.

### 2.3. Lentiviruses Preparation and Generation of Stable Cell Line

The recombinant lentivirus was produced as described previously [25]. Briefly, the full-length murine ICAT cDNA was inserted into the pLent-EF1a-FH-CMV-GFP expression vector and packaged into viral particles in 293T cells with packaging plasmid psPAX2 and envelope plasmid pMD2.G. The virus was collected after 48 h. 3T3-L1 pre-adipocytes were infected with the fresh lentivirus expressing the ICAT for 48 h and the signal of GFP was monitored. Stable 3T3-L1 pre-adipocytes were selected with puromycin for 7 days and sorted through fluorescence activated cell sorting selection (Beckman Coulter, CA, USA). The differentiation of stable pre-adipocytes was induced as the standard protocol. The mRNA and protein expression of ICAT in stable cell line was detected. The lentivirus generated from the empty vector, which expressed only GFP, was used as the control. The cells were also induced into differentiation with the addition of 3.0 μmol/L CHIR99021 to study the role of ICAT on Wnt/β-catenin activity. The pre-adipocytes stably overexpressed E2F1 were also prepared as ICAT.

### 2.4. Transient Expression of ICAT in HeLa and HepG2 Cells

The full-length human ICAT cDNA was sub-cloned by standard PCR into BamHI and EcoRI-restricted pIRES2-EGFP vector. All constructs were verified by DNA sequencing. Transient transfections of Hela and HepG2 cells were performed using Lipofectamine 3000, according to the manufacturer’s instructions. Briefly, 5 μg of pIRES2-EGFP-ICAT vector or empty pIRES2-EGFP vector were transfected into cells cultured at sub-confluent density in 6-well dishes. The signal of GFP was monitored by fluorescence microscope (Zeiss, Germany) 24 h post-transfection. To get the stable ICAT-expressing cell line, Hela and HepG2 cells were maintained in non-selective medium for 2-days post-transfection, then plated in 800 μg/mL G418 medium for 3 weeks, with frequent changes of medium to eliminate dead cells and debris until distinct colonies can be visualized. Individual colonies then are trypsinized and transferred to flasks for further propagation. Stable ICAT overexpressing cells were also sorted by GFP signals through fluorescence activated cell sorting selection, data were analyzed using the Cell Quest software (Beckman Coulter, CA, USA) and expanded for in vitro studies. Cells stably expressed ICAT were grown to 70% confluence on the 6-well plates and treated with 500 μmol/L oleic acid complexed to albumin at a molar ratio of 8:1 for 3 h.

### 2.5. Immunofluorescent Microscopy

Procedures for immunofluorescent staining were essentially the same as previously described. Cells were rinsed twice in PBS, fixed with 4% paraformaldehyde solution for 1 h, permeabilized with 0.1% triton X-100 in PBS for 15 min on ice, blocked with 5% goat serum in PBS for 1 h followed by incubation with primary antibody (1:100, overnight), washed three times with PBS, and incubated with fluorescently labeled secondary antibody (1:50 dilution for 1 h) followed by Hoechst (1:1000). Images for morphological analysis were acquired under a fluorescence microscope.

### 2.6. Quantitative Real-Time PCR

Total RNA was extracted from cells using the Trizol reagent (Aidlab Biotech, Beijing, China). Reverse transcription PCR was performed using the PrimeScript RT Reagent Kit (TaKaRa, Dalian, China) as instructed by the manufacturer and cDNA was used as a template in the subsequent reactions. Real-time PCR was performed using SYBR Premix Ex Taq II (TaKara, Dalian, China) and the ABI-Prism 7500 Sequence Detection System (Applied Biosystems, Foster city, CA, USA) according to the instruction from the manufacturer. The primer sequences (5′–3′) used are listed in Table S1. The mRNA levels of GAPDH and β-actin were used as the internal control. The 2^−ΔΔCt^ method was used to determine the fold changes in mRNA levels of each sample, as compared to the reference sample.

### 2.7. Western Blotting Analysis

Cells were harvested for the analysis of protein abundance by Western blot as previously described [26]. Cells were harvested and lysed on ice for 30 min in RIPA lysis buffer containing 50 mM Tris-HCl (pH 7.4), 150 mmol/L NaCl, 1% NP-40, 0.1% SDS, 1.0 mmol/L Phenylmethanesulfonyl fluoride (PMSF), 1.0 mmol/L Na_3_VO_4_, 1.0 mmol/L NaF, and protease inhibitor tablet (Roche, Indian apolis, IN, USA), followed by sonication for three times with 10 s/ time. The whole-cell lysates were centrifuged at 12,000 rpm for 10 min to collect the supernatant. The protein concentration of the supernatant was determined using the Pierce BCA protein Assay Kit (Huaxing Biotech, Beijing, China) with bovine serum albumin as standard. Equal amounts of proteins were separated using SDS-page gels and transferred to polyvinylidene difluoride (PVDF) membranes (Millipore, MA, USA). The membranes were blocked in 5% fat-free milk in Tris-buffered saline with Tween 20 (TBST) for 1 h at room temperature, and then were incubated with indicated primary antibodies overnight at 4 °C. After incubation with horseradish peroxidase (HRP)-conjugated secondary antibody for 1 h, the chemiluminescent signal was detected using Super-Enhanced Chemiluminescence Kit (Huaxing Biotech, Beijing, China).

### 2.8. Lipid Droplets’ Staining

Lipid droplets in cells were stained by oil red O or BODIPY493/503. Cells were washed with PBS and fixed with 4% paraformaldehyde for 1 h on ice, followed by washing with 60% isopropanol. Then stained with oil red O working solutions containing 6 mL stock solution (5 g/L in isopropanol) and 4 mL double-distilled H_2_O or BODIPY493/503 (stock concentration 1 mg/mL and working solution 1:1000 dilution) for 15 min at room temperature followed by washing for three times with PBS and viewed with a microscope. BODIPY493/503-stained lipid droplets were viewed through a fluorescence microscope. To quantify intracellular lipids, the oil red O-stained lipid droplets were dissolved with 100% isopropanol for 10 min. The absorbance of extracted dye was then measured at 520 nm.

### 2.9. Measurement of TG Content

TG contents in adipocytes were measured using an assay kit as described previously [24]. Briefly, cells were washed and lysed in provided lysis buffer, and then, the TG assay reagents were added, according to the manufacturer’s instructions. The optical density of the solution was measured at 510 nm using a spectrophotometer plate reader. TG contents were calculated from a standard curve for each assay, and data are normalized to total cellular protein contents.

### 2.10. siRNA-Mediated Knockdown

For E2F1 or ICAT knockdown in 3T3-L1 pre-adipocytes, 100 pmol of each siRNA oligonucleotides (Genepharma, Shanghai, China) were transfected into cells plated in 6-well dishes using Lipofectamine RNAiMAX Reagent as described by instructions. Cells transfected with non-targeting siRNA (NC) were used as control. The pre-adipocytes stably overexpressed E2F1 were transfected with 100 pmol siICAT or siNC. Cells were harvested to detect the protein expression or processed for designated assays 48 h post-transfection.

### 2.11. Statistical Analysis

Data were expressed as means ± SEM and were statistically analyzed by GraphPad PRISM5 (GraphPad Software, CA, USA). Both normality and homogeneity tests were examined before the statistical analysis in our study. Differences between two groups were assessed using the unpaired two-tailed Student t test. Data sets that involved more than two groups were assessed using ANOVA, followed by Tukey post-hoc test. In the figures, data with different superscript letters are significantly different at *p* < 0.05. A value according to the post hoc ANOVA statistical analyses. The results were considered statistically significant when *p* < 0.05.

## 3. Results

### 3.1. MDI-Induced Differentiation in 3T3-L1 Cells Was Associated with Increased Protein Levels of E2F1 and ICAT at Day 3 of Differentiation

**Figure 1:**
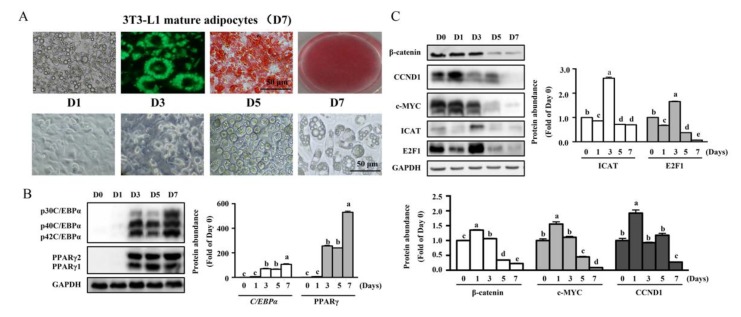
3T3-L1 cell differentiation was associated with an increased protein level of E2F1 and ICAT at day 3 of differentiation. 3T3-L1 pre-adipocytes were differentiated into adipocytes by 1-methyl-3-isobutylxanthine, dexamethasone, and insulin (MDI) medium for 7 days. (**A**) Representative micrographs of the adipocytes during the differentiation process, and adipocytes stained with BODIPY493/503 (green) or oil red O (red). (**B**) Protein levels of PPARγ and C/EBPα during the differentiation progress. (**C**) Protein levels of classic Wnt/β-catenin signaling and E2F1/ICAT during the differentiation. Values are means ± SEMs, *n* = 3 independent experiments. Means without a common letter differ, *p* < 0.05. C/EBPα, CCAAT-enhancer binding protein α; E2F1, E2 promoter binding factor 1; GAPDH, glyceraldehyde-3-phosphate dehydrogenase; ICAT, Inhibitor of β-catenin and TCF4; PPARγ, peroxisome proliferator activated receptor γ.

In consistency with the previous study [19], 3T3-L1 pre-adipocytes were successfully differentiated into adipocytes by MDI medium with the appearance of marked multiple vesicles and lipid accumulation as shown by oil red O and BODIPY493/503 staining (Figure 1A, upper lane). The representative micrographs of cells during differentiation showed that accumulation of the lipid droplets was observed at day 3 (Figure 1A, lower lane) and differentiated into mature adipocytes with 7-day MDI induction. The time course study showed that transcriptional (Figure S1A) and protein levels of PPARγ and C/EBPα (Figure 1B), two critical adipogenic regulators, were significantly enhanced (*p* < 0.05). Both the mRNA level (Figure S1B) and protein abundance of β-catenin, as well as these of c-MYC and CCND1 (Figure 1C), two classic downstream targets of Wnt/β-catenin signaling, were dramatically downregulated (*p* < 0.05) in differentiated cells, as compared with un-differentiated cells. In agreement with the phenotype changes, mRNA level of fatty acid binding protein (AP2), a well-known adipocyte marker, was upregulated (*p* < 0.05) (Figure S1A, lower panel). Of interest, protein levels of E2F1 and ICAT were significantly increased (*p* < 0.05) at day 3 of differentiation and were reduced to an undetectable level at the later stages of adipocyte differentiation (Figure 1C). These results showed that MDI-induced differentiation in 3T3-L1 cells was associated with an increased protein level of E2F1/ICAT at day 3 of differentiation.

### 3.2. Activation of Wnt/β-catenin Signaling by GSK3β Inhibitor Blocked Adipogenesis

**Figure 2:**
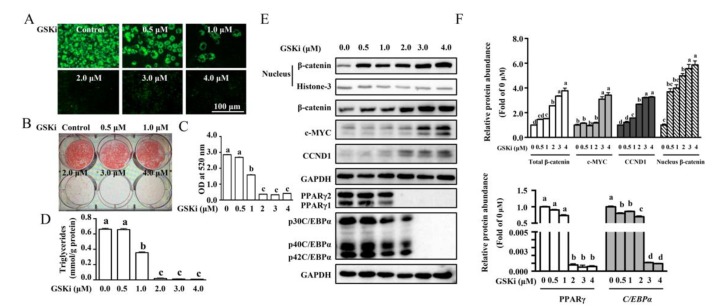
Activation of Wnt/β-catenin signaling by GSK3β inhibitor abolished MDI-induced adipogenesis. 3T3-L1 cells were induced to differentiation in the presence of CHIR99021 (0, 0.5, 1.0, 2.0 3.0, or 4.0 μmol/L) for 7 days. (**A**) Representative micrographs of mature adipocytes stained with BODIPY493/503 (green) and (**B**) plate photograph of adipocytes stained with oil red O (red). (**C**) Lipid accumulation and (**D**) triglyceride (TG) contents of 3T3-L1 adipocytes after 7-day CHIR99021 induction. (**E**) Protein expression and (**F**) abundance analysis for Wnt/β-catenin signaling and adipogenic regulators. Values are means ± SEMs, *n* = 3 independent experiments. Means without a common letter differ, *p* < 0.05. C/EBPα, CCAAT-enhancer binding protein α; GSKi, CHIR99021; GAPDH, glyceraldehyde-3-phosphate dehydrogenase; PPARγ, peroxisome proliferator activated receptor γ.

To further explore a functional role of Wnt/β-catenin signaling on differentiation, 3T3-L1 cells were incubated with MDI to induce differentiation in the presence of CHIR99021 (0, 0.5, 1.0, 2.0, 3.0, or 4.0 μM), a GSK3β inhibitor, which has been reported to activate the canonical Wnt/β-catenin pathway in 3T3-L1 pre-adipocytes [22]. Adipogenesis was assessed at day 7 and we found that CHIR99021 blocked 3T3-L1 differentiation in a dose-dependent manner, as assessed by oil red O and BODIPY493/503 staining (Figure 2A,B). Quantification of lipid accumulation (Figure 2C) and intracellular TG (Figure 2D) indicated that differentiation of pre-adipocytes was significantly inhibited by the presence of 1.0 to 4.0 μM CHIR99021 in the media. CHIR99021-activated Wnt/β-catenin signaling was validated by an increased protein level of β-catenin in the nucleus, as well as upregulated proteins abundance of CCND1 and c-MYC (*p* < 0.05) (Figure 2E,F) in 3T3-L1 adipocytes. In agreement with phenotypes observed and activation of Wnt/β-catenin signaling, 3T3-L1 adipocytes incubated with 3.0 and 4.0 μM CHIR99021 led to significantly reduced protein abundance of PPARγ and C/EBPα (*p* < 0.05) (Figure 2E,F). In our study, 3.0 μM of CHIR99021 was chosen for the further research, considering a markedly repressing effect on pre-adipocyte differentiation.

### 3.3. Overexpression of ICAT Reversed the Effect of GSK3β Inhibitor on Cell Differentiation and Adipogenesis in 3T3-L1 Pre-Adipocytes

**Figure 3:**
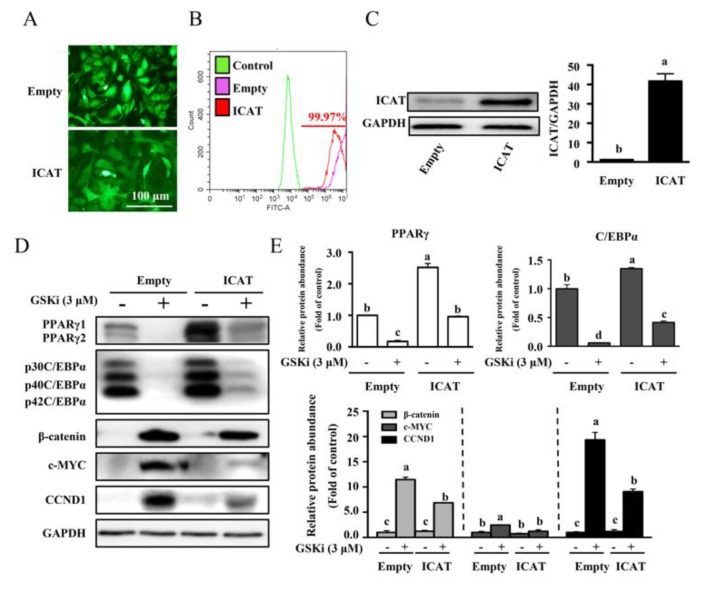
ICAT overexpression inhibited the Wnt/β-catenin signaling targets, but upregulated protein levels of adipogenic regulators upon the CHIR99021 induction. (**A**) 3T3-L1 pre-adipocytes infected with lentivirus carrying ICAT. (**B**) 3T3-L1 pre-adipocytes stably overexpressing ICAT sorted by flow cytometry. (**C**) Protein expression for ICAT in stable overexpressing cells. (**D**) Protein expressions and (**E**) abundance analysis for adipogenic regulators and Wnt/β-catenin targets in 3T3-L1 adipocytes after 7 days differentiation. Values are means ± SEMs, *n* = 3 independent experiments. Means without a common letter differ, *p* < 0.05. C/EBPα, CCAAT-enhancer binding protein α; GSKi, CHIR99021; GAPDH, glyceraldehyde-3-phosphate dehydrogenase; ICAT, Inhibitor of β-catenin and TCF4; PPARγ, peroxisome proliferator activated receptor γ.

**Figure 4:**
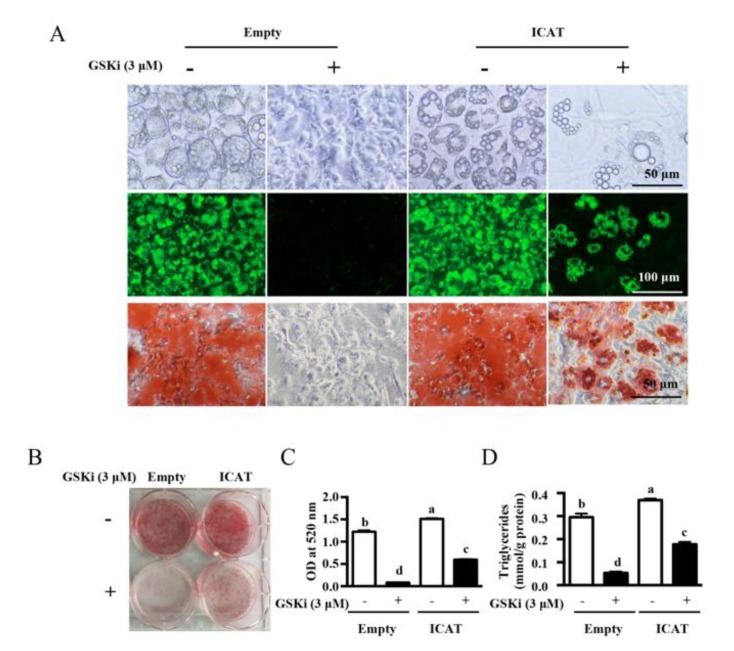
Overexpression of ICAT reversed the effect of GSK3β inhibitor on cell differentiation and adipogenesis in 3T3-L1 adipocytes. Cells were treated as in Figure 3 and were collected after 7-day differentiation. (A) Representative micrographs of adipocytes stained with or without BODIPY493/503 (green) and oil red O (red). (B) Photograph of adipocytes stained with oil red O (red). (C) Lipid contents and (D) TG contents of 3T3-L1 adipocytes. Values are means ± SEMs, *n* = 3 independent experiments. Means without a common letter differ, *p* < 0.05. GSKi, CHIR99021; ICAT, Inhibitor of β-catenin and TCF4.

To investigate a functional role of ICAT on adipogenesis, 3T3-L1 pre-adipocytes were transfected with ICAT expression vector using a lentivirus-mediated transfection method. The transfection efficiency was confirmed at 48 h post-infection (Figure 3A) and almost all pre-adipocytes were GFP positive after 7-day selection by puromycin (Figure 3B). As expected, lentivirus infection resulted in marked upregulation of ICAT at both mRNA (Figure S2A) and protein levels (Figure 3C) (*p* < 0.05). To identify a repressing effect of ICAT on Wnt/β-catenin activity in 3T3-L1 pre-adipocytes, the pre-adipocytes stably overexpressed ICAT or the empty vector were treated with or without CHIR99021, a GSK3β inhibitor. As shown, Western blot analysis showed that CHIR99021 treatment led to remarkable upregulation of β-catenin and its downstream targets, including c-MYC and CCND1, as well as significantly downregulation of protein abundances for PPARγ and C/EBPα (*p* < 0.05) (Figure 3D,E). These effects of CHIR99021 were partial revered by ICAT overexpression. In agreement with protein expression, we found that MDI-induced differentiation was markedly inhibited by CHIR99021 treatment, as shown by oil red O and BODIPY493/503 staining (Figure 4A,B), as well as quantification of lipid accumulation (Figure 4C) and intracellular TG (*p* < 0.05) (Figure 4D). Interestingly, a repressing effect of GSK3β inhibitor CHIR99021 on differentiation, TG accumulation was partially reversed by ICAT overexpression in 3T3-L1 cells (Figure 4). Although ICAT overexpression had a modest repressive effect on the protein level of β-catenin, the protein levels of c-MYC and CCND1, two downstream targets of β-catenin, were remarkable downregulated, while those of PPARγ and C/EBPα (Figure 3D,E), as well as CHIR99021-induced repressing effect on differentiation were significantly reversed by ICAT (Figure 4). This finding indicates that ICAT repressed the β-catenin-TCF4-mediated transactivation instead of directly downregulating the β-catenin expression in the adipocytes in our study.

### 3.4. ICAT or E2F1 Knockdown Inhibited MDI-Induced Differentiation of Pre-Adipocytes

**Figure 5:**
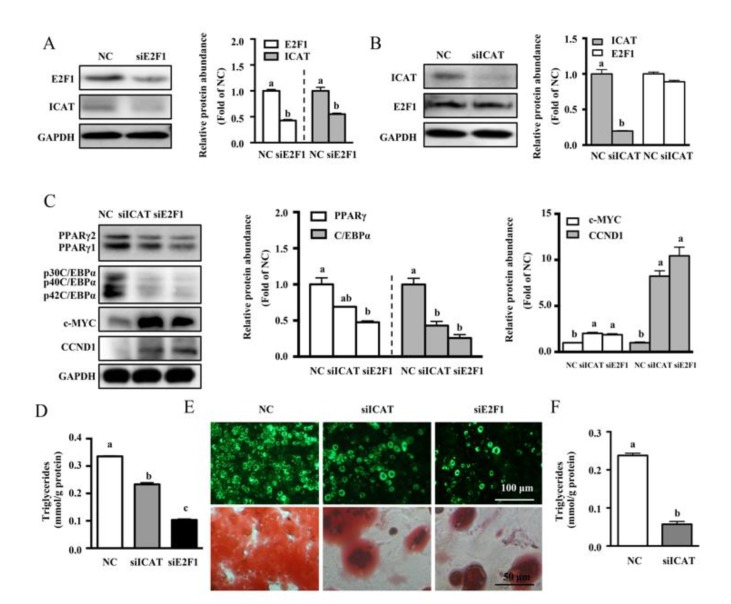
ICAT or E2F1 knockdown inhibited MDI-induced differentiation of pre-adipocytes. (**A**) Protein levels for E2F1 and ICAT at 48 h after siRNA-mediated knockdown of E2F1. (**B**) Protein levels for ICAT and E2F1 at 48 h after siRNA-mediated knockdown of ICAT. (**C**) Protein abundances for adipogenic regulators and Wnt/β-catenin targets, and (**D**) TG contents of 3T3-L1 adipocytes after ICAT or E2F1 knockdown. (**E**) Representative micrographs of siE2F1 or siICAT transfected adipocytes after BODIPY493/503 (green) or oil red O (red) staining. (**F**) TG contents of stable E2F1 overexpressed 3T3-L1 adipocytes after ICAT knockdown. Values are means ± SEMs, *n* = 3 independent experiments. Means without a common letter differ, *p* < 0.05. C/EBPα, CCAAT-enhancer binding protein α; E2F1, E2 promoter binding factor 1; GAPDH, glyceraldehyde-3-phosphate dehydrogenase; ICAT, Inhibitor of β-catenin and TCF4; PPARγ, peroxisome proliferator activated receptor γ.

ICAT has been reported to be a direct target of E2F1 that is responsible for the suppression of β-catenin activity in cancer cells [17]. To test an involvement of E2F1/ICAT on cell differentiation and adipogenesis, 3T3-L1 cells were transfected with siRNA targeting E2F1 or ICAT. As shown, siRNA-mediated knockdown of E2F1 in 3T3-L1 cells resulted in a marked decrease of E2F1 and ICAT at protein level (*p* < 0.05) (Figure 5A), confirming the regulation of ICAT expression by the endogenous E2F1 in 3T3-L1 cells. ICAT siRNA transfection led to a reduced protein level of ICAT (*p* < 0.05) without affecting that of E2F1 (Figure 5B), validating an efficiency of siRNA in 3T3-L1 pre-adipocytes. Then, cells transfected with non-targeting siRNA (NC), siCAT, or siE2F1 were incubated with MDI to evaluate whether ICAT or E2F1 is functionally required for adipogenesis in 3T3-L1 cells. We found that differentiation induced by MDI was markedly inhibited in siE2F1 or siICAT transfection in 3T3-L1 pre-adipocytes. In addition, MDI-induced increased TG contents (Figure 5D) and lipid accumulation (Figure 5E) were dramatically reduced (*p* < 0.05) by gene silencing of E2F1 or ICAT. Western blot analysis showed that protein levels of C/EBPα and PPARγ were reduced, while those of c-MYC and CCND1 were upregulated (*p* < 0.05) (Figure 5C) by siE2F1 or siIACT transfection in 3T3-L1 cells. Compared with siE2F1 transfected cells, ICAT knockdown had a modest inhibitory effect on differentiation (Figure 5D,E). The TG contents in the stable E2F1 overexpressed adipocytes were markedly reduced (*p* < 0.05) by gene silencing of ICAT (Figure 5F), confirming the ICAT was required for E2F1 during differentiation.

### 3.5. ICAT Did Not Affect Lipid Accumulation in Hela and HepG2 Cells

**Figure 6:**
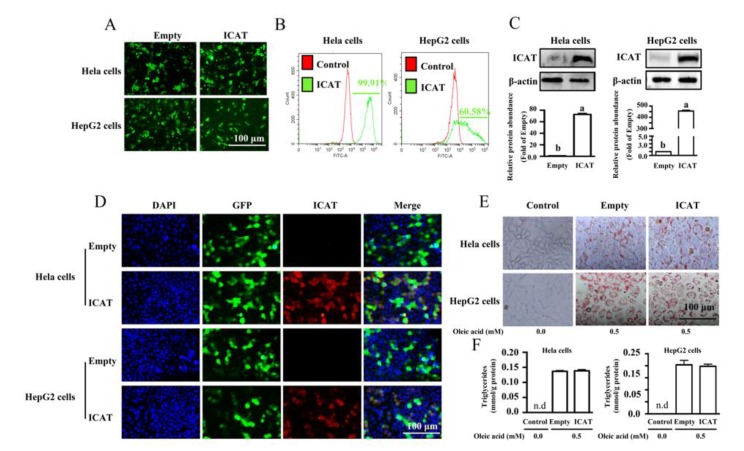
ICAT did not affect lipid accumulation in Hela and HepG2 cells. (**A**) The transfection efficiency and (**B**) purity of Hela and HepG2 cells stably expressed ICAT. (**C**) Protein levels and (**D**) in situ expressions of ICAT in both cell lines. (**E**) Representative micrographs of cells stained with oil red O. (**F**) TG contents of Hela and HepG2 after 0.5 mmol/L oleic acid treatment. Values are means ± SEMs, *n* = 3 independent experiments. Means without a common letter differ, *p* < 0.05. ICAT, Inhibitor of β-catenin and TCF4.

Hela and HepG2 cells can directly accumulate lipid droplets in the cells with the induction of oleic acid [27,28,29]. Importantly, these characteristics of lipid droplets fusion and degradation in the Hela and HepG2 cells are shared by mature adipocytes, therefore, these two cell lines are used as cell line models in studies related to lipid droplets turnover during the lipogenesis and lipolysis processes [27,28,29]. To investigate an effect of ICAT on oleic acid-induced lipid accumulation, Hela and HepG2 cells were transfected with pIRES2-EGFP-ICAT expression vector or empty vector. The transfection efficiency of cells (Figure 6A) and purity of cells stably expressed ICAT was confirmed (Figure 6B). The mRNA level of ICAT in cells after G418 selection was confirmed (Supplemental Figure S2B,C). The protein level (Figure 6C) and in situ expression of ICAT (Figure 6D) was validated by Western blot analysis and immunofluorescence staining in both cell lines. Oleic acid treatment led to increased TG contents as shown by oil red O staining in Hela and HepG2 cells (Figure 6E). ICAT overexpression did not affect oleic acid-induced lipid accumulation in both cell lines (Figure 6F). These data indicated that ICAT did not implicate in lipid accumulation at the surface of Hela and HepG2 cells.

## 4. Discussion

**Figure 7:**
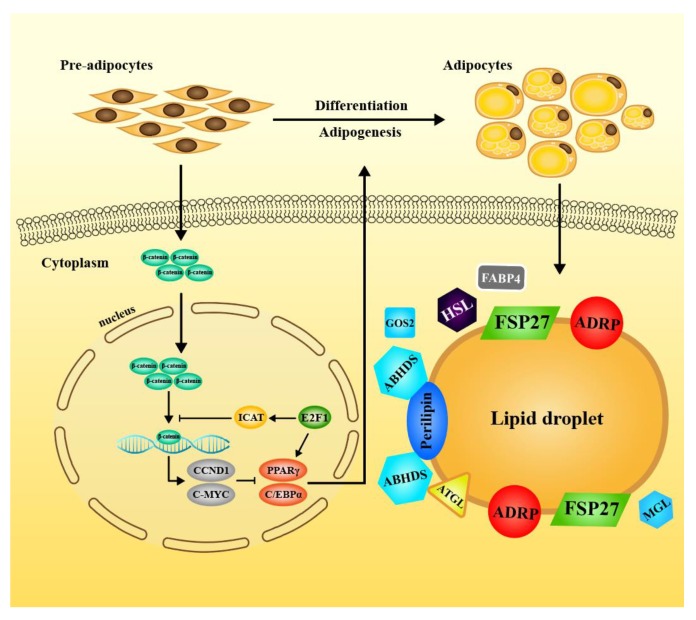
Proposed model on crosstalk between E2F1/ICAT and Wnt/β-catenin signaling on the differentiation of pre-adipocytes. The stabilized free cytosolic β-catenin translocate to the nucleus to interaction with the TCF4 for activation of the Wnt/β-catenin targets and inhibit adipogenesis by blocking induction of C/EBP and PPAR proteins. The activating ICAT by E2F1 competes with the β-catenin to repress the β-catenin-TCF4-mediated transactivation instead of directly downregulating the β-catenin expression, and reverses the inhibitory effect of Wnt/β-catenin signaling on MDI-induced adipogenesis. ICAT activation downregulates the protein expression of c-MYC and CCND1 and abolishes their suppression on protein expression of C/EBPα and PPARγ, thus functioning as a promotive regulator for adipogenesis during differentiation.

In the present study, we found a novel function of E2F1/ICAT on promoting pre-adipocytes differentiation and lipid accumulation in 3T3-L1 cells. Depletion of E2F1 or ICAT by siRNA led to reduced differentiation and decreased TG contents by inhibiting expression of C/EBPα and PPARγ in MDI-treated cells. Overexpression of ICAT in 3T3-L1 pre-adipocytes markedly promoted the adipogenesis induced by MDI. In addition, this promotive effect was also observed in CHIR99021-treated cells, in which Wnt/β-catenin was activated by inhibiting GSK3β, a negative regulator of Wnt/β-catenin signaling, therefore repressing differentiation of pre-adipocytes. An inhibitory effect of CHIR99021 on adipogenesis was partially attenuated by lentivirus-mediated ICAT overexpression in 3T3-L1 cells. In addition, the adipogenesis in the stable E2F1 overexpressed adipocytes were markedly reduced by ICAT knockdown. This effect of E2F1/ICAT on differentiation was mainly mediated by regulating Wnt/β-catenin signaling. These findings revealed a crosstalk between E2F1/ICAT signaling and Wnt/β-catenin on pre-adipocyte differentiation (Figure 7).

Adipogenesis is a critical process implicated in the development of metabolic disease. Both clinical and experimental data have indicated that small molecules or compounds with the ability to interfere in the adipogenesis process might be a potentially therapeutic agent for obesity [30,31]. Considering that the number of fat cells in the body is relatively stable in adults [32], it is of great significance to reveal underlying mechanisms responsible for pre-adipocytes differentiation and adipogenesis. Wnt/β-catenin is a crucial repressor for adipogenesis, whose activity is inhibited during pre-adipocytes differentiation [22]. In response to extracellular stimulations, the canonical Wnt/β-catenin signaling is activated and leads to stabilization of β-catenin in the cytoplasm, accumulated β-catenin translocated to the nucleus, and interacts with T-cell factor/ lymphoid enhancer-binding factor (TCF/LEF) to trans-activate downstream targets, such as CCND1, c-MYC, PPARγ, and C/EBPα, therefore regulating cellular metabolism [33,34]. Consistently, it has been reported that some Chinese herbal medicines, such as curcumin, shikonin, and resveratrol, regulate metabolic homeostasis by regulating Wnt/β-catenin signaling pathway [35,36,37].

ICAT has been identified as a direct transcriptional target of E2F1 [17], through which E2F1 interacts with Wnt/β-catenin and regulates proliferation in colon cancers [7,17,38,39]. In addition to a well-known function in tumorigenesis of humans, E2F1 has been described as a transcription factor participates in the development of multiple metabolic diseases, including obesity, diabetes, and fatty liver disease [10,12,14]. An in vitro study has shown that induction of E2F1 during the early phase of differentiation is critical for adipogenesis [7]. However, mechanistic insight responsible for this regulation remains largely unknown.

In the present study, we found that the protein levels of E2F1 were reduced at 24 h treatment, which was dramatically enhanced on the day 3 of differentiation, and then reduced to an undetectable level as the cell differentiated into mature adipocytes. This result was consistent with a previous study [14]. The shift of E2F1 during the differentiation is dependent on its function in cell proliferation and differentiation. Before induction of differentiation, 3T3-L1 pre-adipocytes was proliferated and reached confluency, therefore, protein level of E2F1 was higher as seen at day 0. After that, 3T3-L1 preadipocytes were growth-arrested and the proliferative effect of E2F1 is repressed by repressive regulator of cell cycle proliferation program, and leading to a reduced protein level at day 1. After being treated with MDI for 72 h, the activity of E2F1 was activated to facilitate the adipogenesis during differentiation as shown in Figure 1C. Of interest, the protein level of E2F1 was reduced to an undetectable level, indicating a requirement of E2F1 for initiation of differentiation, whose activity was not needed at the later stages of adipocyte differentiation [7,14]. In addition, we observed a similar expression profile on the protein level of ICAT, followed by the induction of PPARγ and C/EBPα, as well as decreased protein level of β-catenin, indicating a potential implication of E2F1/ICAT in adipogenesis.

Adipogenesis is associated with decreased activation of β-catenin signaling. To investigate a promotive effect of ICAT on adipogenesis by repressing β-catenin, 3T3-L1 pre-adipocytes transfected with ICAT or empty vector were treated with GSK3β inhibitor to upregulate β-catenin. As expected, GSK3β inhibitor treatment led to enhanced β-catenin and blocked cell differentiation. This effect was partially reversed by ICAT overexpression as shown by alterations in lipid droplets and TG contents. PPARγ and C/EBPα are critical regulators for cell differentiation, which were negatively regulated by CCND1 and c-MYC, respectively [40,41,42], through which β-catenin exert a regulatory effect on adipogenesis [43]. ICAT overexpression reversed GSK3β inhibitor-induced phenotype alteration as well as protein levels of adipogenic regulators, therefore exerting a promotive effect on differentiation. Importantly, overexpression of ICAT in 3T3-L1 pre-adipocytes also markedly promoted the adipogenesis compared to the control cells during differentiation. Moreover, MDI-induced differentiation was attenuated in siE2F1 or siICAT transfected cells and protein expressions of Wnt/β-catenin targets including c-MYC and CCND1 were increased, solidifying a promotive effect of endogenous E2F1/ICAT on differentiation by repressing Wnt/β-catenin signaling. It has been reported that ICAT inhibit the interaction of β-catenin with TCF4, leading to the repression of β-catenin-TCF4-mediated transactivation [18]. Our previous study about the TCF4-dependent luciferase reporter activity also confirmed that ICAT was a robust inhibitor of β-catenin/TCF4 activity [17]. It is worthwhile to note that ICAT overexpression reversed GSK3β inhibitor-induced phenotype alteration to promote differentiation and downregulated the c-MYC and CCND1 without affecting the accumulation of β-catenin induced by the GSKi in the nucleus, indicating that ICAT repressed the β-catenin-TCF4-mediated transactivation instead of directly downregulating its protein level. Furthermore, ICAT knockdown inhibited the adipogenesis in the stable E2F1 overexpressed adipocytes, confirming the ICAT was required for E2F1 during differentiation. These findings demonstrated that ICAT is a novel target that is responsible for E2F1′s regulation for pre-adipocyte differentiation by interacting with Wnt/β-catenin signaling.

Of note, we found that siE2F1 transfected cells had a higher inhibitory effect on MDI-induced differentiation, as compared with siICAT transfected cells. This result is due to the following reasons. First, besides ICAT as described herein, E2F1 can regulate pre-adipocytes differentiation by inducing other targets, such as Perp-1 [44,45] and PPAR [46], and contribute to lipid accumulation in 3T3-L1 cells [47]. Second, E2F1 can regulate cell cycle progress in multiple cells, knockdown of E2F1 might affect adipogenesis due to its interfering effect on cell proliferation as previously described [48,49]. Notably, ICAT overexpression did not affect oleic acid-induced lipid accumulation in cell membrane of Hela and HepG2 cells. It has been reported that Wnt/β-catenin signaling is not implicated in oleic acid-induced lipid accumulation [50,51], therefore, it is plausible that ICAT did not act in this model. The findings indicated that E2F1/ICAT mainly regulated initiation of pre-adipocytes differentiation at early stage and led to lipid accumulation, without affecting lipid content in mature adipocytes. These data indicated a specific effect of E2F1/ICAT on differentiation and lipid accumulation in pre-adipocytes. It has been reported that protein level E2F1 is markedly reduced in later stage of differentiation, but the reason is not known. Another novel finding of our study is we showed, for the first time, that E2F1/ICAT is critical for the maintenance of PPARγ and C/EBPα by blocking the c-MYC and CCND1, two negative regulators, such as PPARγ and C/EBPα, and contributing to the progress of differentiation.

It has been reported that E2F1 transcriptional activity is enhanced in obese subjects and non-alcoholic fatty liver disease [52]. Our data provided herein indicated that E2F1 might promote adipogenesis by ICAT-mediated inhibition on β-catenin. Of note, E2F1 is a transcriptional factor with multiple functions, including cell proliferation, differentiation, and apoptosis [6]. Depletion of E2F1 might affect adipogenesis as well as other biological processes, and lead to potentially deleterious effects in both humans and animals [53]. Considering that ICAT is required for E2F1′s activity on pre-adipocyte differentiation, ICAT might be a therapeutic target for metabolic diseases, especially the patients with enhanced protein level of E2F1 and ICAT.

## Supplementary Materials

The following are available online at https://www.mdpi.com/2073-4409/9/4/1024/s1, Table S1: Primers for RT-PCR. Figure S1: mRNA expressions of adipogenic regulators were enhanced, while Wnt/β-catenin targets were reduced during the differentiation process. Figure S2: Gene expressions of ICAT in 3T3-L1, Hela and HepG2 cells.
