# Supplementary material for: E2F1 Regulates Adipocyte Differentiation and Adipogenesis by Activating ICAT
Source: Cells. 2020 Apr 21;9(4):1024. doi: 10.3390/cells9041024 (PMC7225968; doi:10.3390/cells9041024)
Supplement: Supplementary file 1 [file cells-09-01024-s001.zip › Supplementary materials/Supplementary materials.pdf]

Table S1 Primers for RT-PCR

Gene name	Primer sequence (5'-3')
m-C/EBPα	F: AGGTGCTGGAGTTGACCAGT<br>R: CAGCCTAGAGATCCAGCGAC
m-CCND1	F: CACAACGCACTTTCTTTCCA<br>R: ACCAGCCTCTTCCTCCACTT
m-GAPDH	F: AAGCCCATCACCATCTTCCA<br>R: CACCAGTAGACTCCACGACA
m-c-MYC	F: GCTCGCCCAAATCCTGTA<br>R: AGGACTCGGAGGACAGCA
m-β-catenin	F: GCTGCTCATCCCACTAATGT<br>R: CCGCGTCATCCTGATAGTTAAT
m-ICAT	F: CAGTGAGGAGGAATTTCTGCG<br>R: ATCACCACGTCCTCTGCACC
m-PPARγ	F: AGGCGAGGGCGATCTTGACAG<br>R: AATTCGGATGGCCACCTCTTTG

m-AP2	F: TGAAGAGCATCATAACCCTA<br>R: TCATAACACATTCCACCACC
h-GAPDH	F: CCAAGGAGTAAGACCCCTGG<br>R: AGGGGAGATTCAGTGTGGTG
h-ICAT	F: CCCTTCAGACTGGCCCTTAA<br>R: CCCTTCAGACTGGCCCTTAA

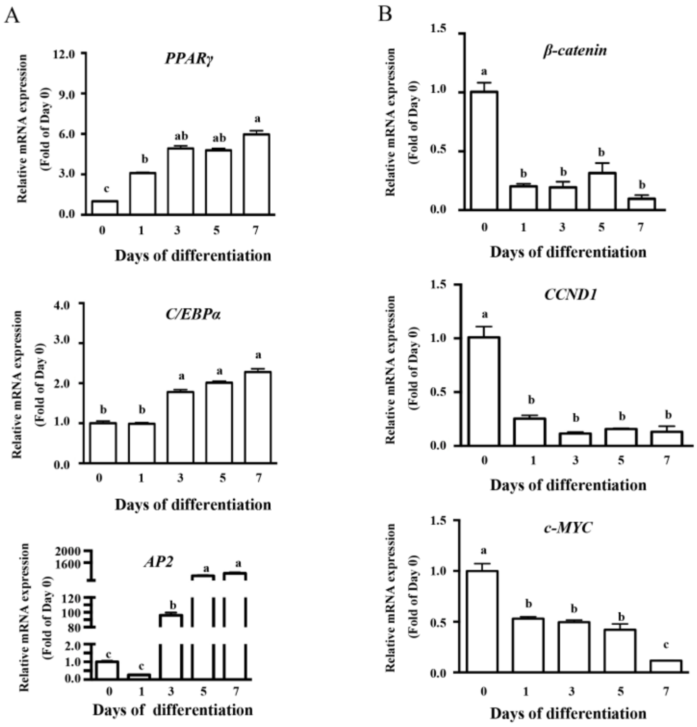

Figure S1. mRNA expressions of adipogenic regulators were enhanced, while Wnt/β-catenin targets were reduced during the differentiation process. 3T3-L1 pre-adipocytes were

differentiated into adipocytes by MDI medium for 7 days. (A) mRNA levels of the adipogenic regulators during the differentiation progress. (B) mRNA levels of the Wnt/β-catenin targets during the differentiation progress. Values are means ± SEMs, $n = 3$ independent experiments. Means without a common letter differ, $P < 0.05$. AP2, fatty acid binding protein; C/EBPα, CCAAT-enhancer binding protein α; PPARγ, peroxisome proliferator activated receptor γ.

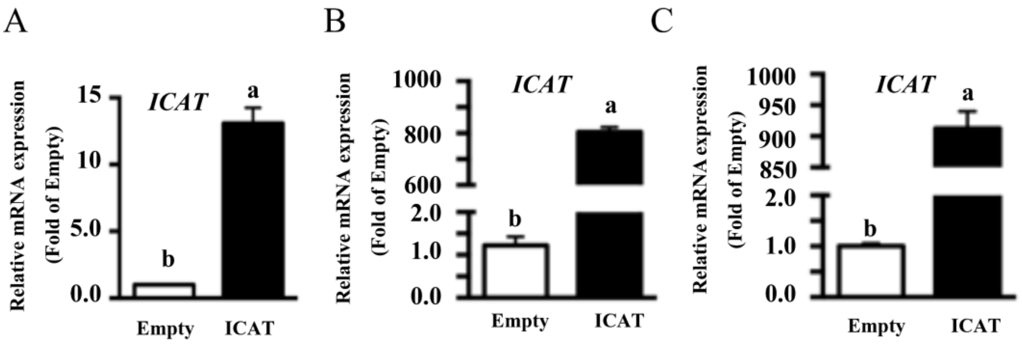

Figure S2. Gene expressions of ICAT in 3T3-L1, Hela and HepG2 cells. (A) Gene levels of ICAT in stable overexpressed 3T3-L1 cells, (B) Hela cells and (C) HepG2 cells. Values are means ± SEMs, $n = 3$ independent experiments. Means without a common letter differ, $P < 0.05$. ICAT, Inhibitor of β-catenin and TCF4.
