# Supplementary figures and images for: E2F1 Regulates Adipocyte Differentiation and Adipogenesis by Activating ICAT
Source: Cells. 2020 Apr 21;9(4):1024. doi: 10.3390/cells9041024 (PMC7225968; doi:10.3390/cells9041024)

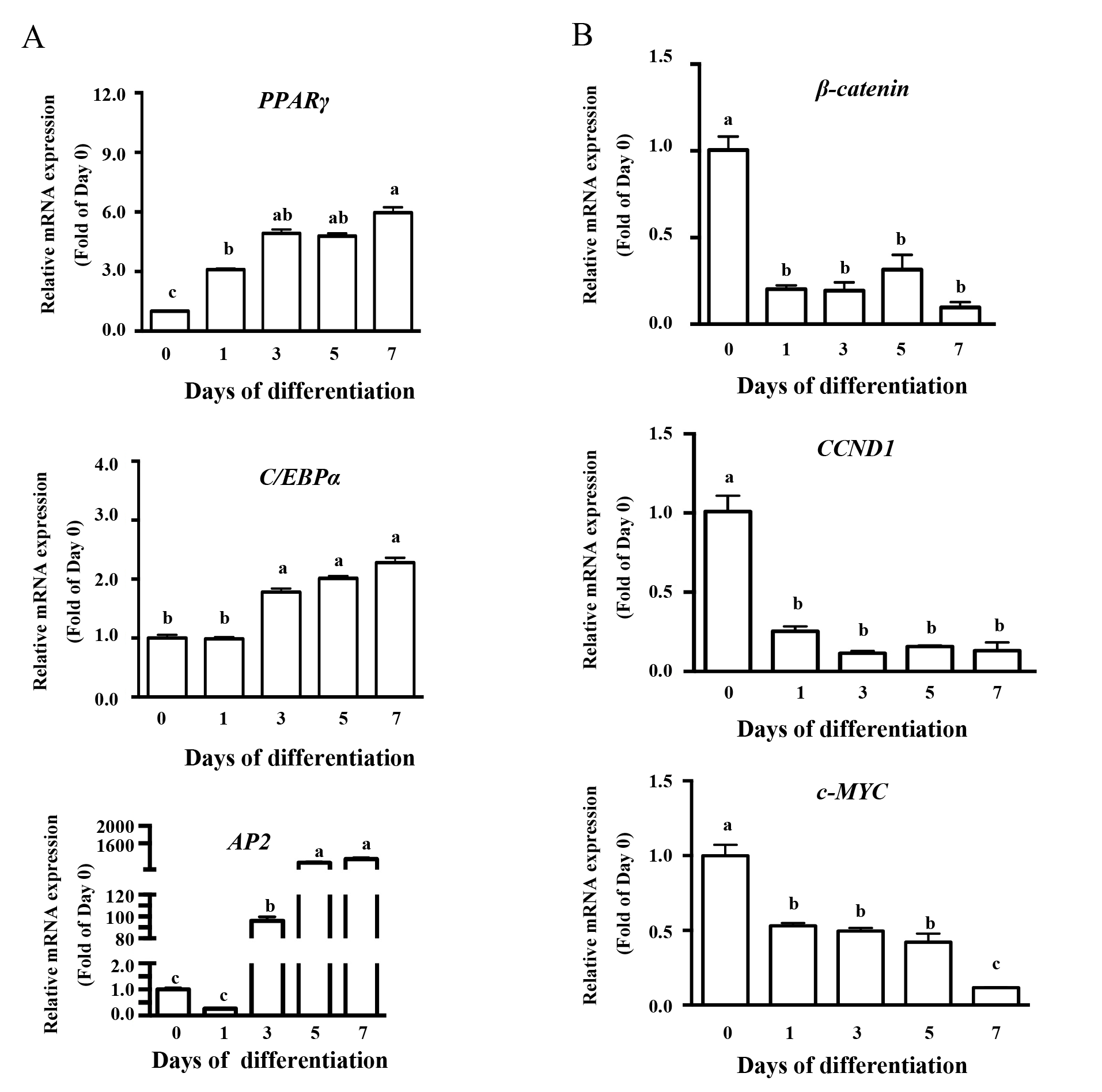

Supplement: Supplementary file 1 [file cells-09-01024-s001.zip › Supplementary materials/Figure S1.tif]

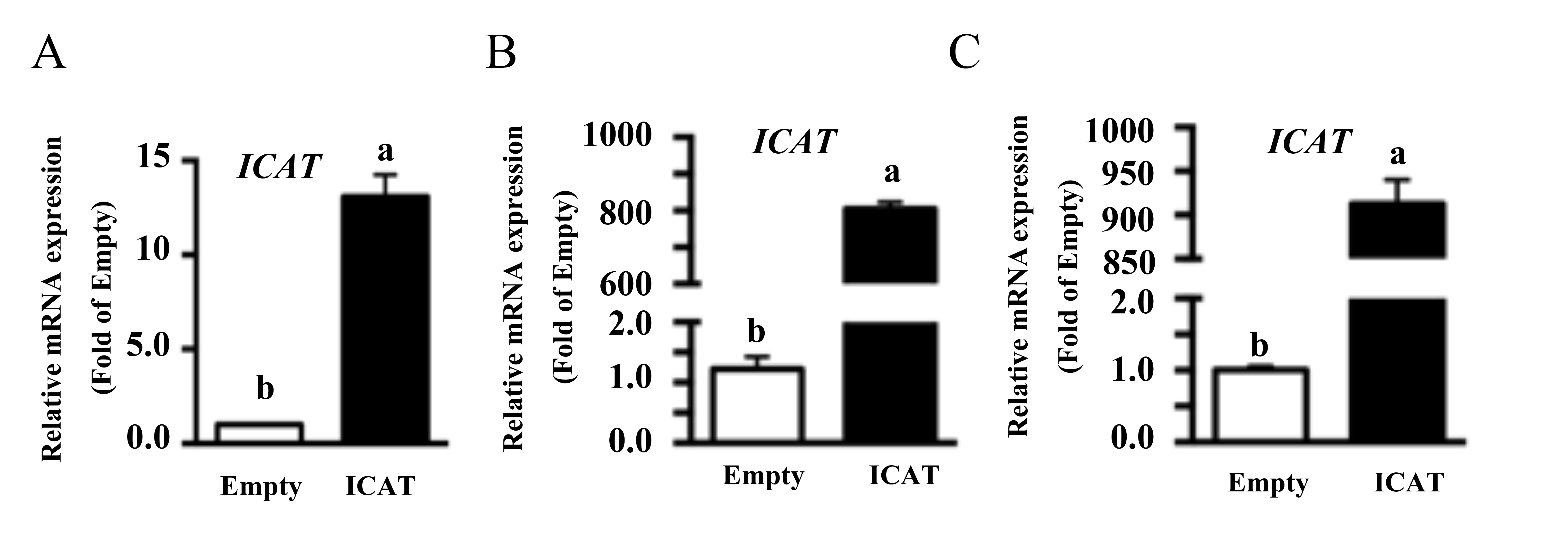

Supplement: Supplementary file 1 [file cells-09-01024-s001.zip › Supplementary materials/Figure S2.tif]
